# Corticosterone Contributes to Context‐Triggered Retrieval of Morphine Withdrawal Memories by Acting on Basolateral Amygdala Neurons Projecting to Nucleus Accumbens Core

**DOI:** 10.1002/advs.202503409

**Published:** 2025-08-23

**Authors:** Zixuan Cao, Yaxian Wen, Yuanqi Chen, Yali Fu, Hao Yang, Chenshan Chu, Xinli Guo, Yu Yuan, Chao Lei, Huan Sheng, Da Shao, Li Yang, Dongyang Cui, Ming Chen, Bin Lai, Ping Zheng

**Affiliations:** ^1^ State Key Laboratory of Medical Neurobiology Institutes of Brain Science MOE Frontier Center for Brain Science Department of Neurology of Zhongshan Hospital Fudan University Shanghai 200032 China

**Keywords:** BLA^→NAcC^ projection neurons, corticosterone, D1 receptors, glucocorticoid receptors, mineralocorticoid receptors, morphine withdrawal memories retrieval

## Abstract

Context‐triggered retrieval of drug withdrawal memories (CTR‐DWM) is a major cause of drug relapse. Most studies of the context‐triggered retrieval of morphine withdrawal memories (CTR‐MWM) have mainly focused on the functional interactions within the central structures of the brain. It remains unknown how an increase in corticosterone, which is an important response under drug withdrawal state, participates in CTR‐MWM. The present results show that corticosterone contributes to CTR‐MWM; within the basolateral amygdala (BLA), it is the mineralocorticoid receptor (MR), rather than the glucocorticoid receptor (GR), activated by corticosterone that mediates CTR‐MWM; MR of BLA neurons projecting to the nucleus accumbens core (BLA^→NAcC^) mediates CTR‐MWM; MR increases presynaptic glutamate release and participates in dopamine D1 receptor ‐induced increase in presynaptic glutamate release and postsynaptic AMPA (α‐Amino‐3‐hydroxy‐5‐methyl‐4‐isoxazolepropionic Acid) currents; MR increases intrinsic excitability of BLA^→NAcC^ neurons during CTR‐MWM. These results suggest that corticosterone contributes to CTR‐MWM by activating BLA^→NAcC^ neurons through MR pathways, uncovering a link between a systemic hormonal response and a specific CTR‐MWM process.

## Graphical abstract

Context associated with morphine withdrawal elicits an increase in serum corticosterone levels. Corticosterone participates in CTR‐MWM by acting on MR, but not GR, in the BLA. MR in BLA^→NAcC^ neurons mediates CTR‐MWM. MR increases presynaptic glutamate release and meanwhile participates in D1 receptor‐induced increases in presynaptic glutamate release and postsynaptic AMPA currents. MR increases the intrinsic excitability of BLA^→NAcC^ neurons during CTR‐MWM.

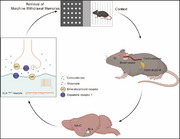

## 1 Introduction

Drug addiction represents a persistent brain disorder characterized by an uncontrolled urge to seek drugs and a high propensity for relapse.^[^ ^1^ ^]^ Conditioned context through the association with drug withdrawal experience is capable of triggering the retrieval of drug withdrawal memories and thus leads to drug relapse.^[^ ^2^, ^3^ ^]^ Therefore, a better understanding of the mechanism underlying conditioned context‐triggered retrieval of drug withdrawal memories (CTR‐DWM) is important for the development of therapies for drug relapse.

A commonly utilized animal model for investigating the mechanism behind CTR‐DWM is conditioned place aversion (CPA).^[^ ^4^, ^5^, ^6^ ^]^ Using this model, previous studies found that conditioned context could activate multiple regions of brain, including the nucleus accumbens (NAc),^[^ ^7^ ^]^ the hippocampus,^[^ ^8^ ^]^ the paraventricular nucleus of the thalamus (PVT),^[^ ^9^ ^]^ the basolateral amygdala (BLA),^[^ ^10^, ^11^, ^12^ ^]^ to trigger the retrieval of drug withdrawal memories. However, it remains poorly understood whether the context mobilizes hormones to be involved in the CTR‐DWM.

Corticosterone is a crucial hormone mediating the body's response to stressors.^[^ ^13^ ^]^ Negative effects and increased anxiety associated with the acute drug withdrawal stage are also stressors that can elicit an increase in corticotropin‐releasing hormone and corticosterone via activating the hypothalamo‐pituitary‐adrenal axis (HPA axis).^[^ ^14^, ^15^, ^16^, ^17^ ^]^ Therefore, when drug withdrawal symptoms occur, it is possible that environmental context establishes an association with corticosterone, and thus the originally neutral context may acquire the ability to elicit an elevation in corticosterone. Studies have revealed a critical role of corticotropin‐releasing hormone (CRH) and CRH1 receptor in the negative affective‐like states of drug withdrawal.^[^ ^14^, ^15^ ^]^ Likewise, the role of corticosterone in the withdrawal stage has been increasingly explored. For example, Lin et al. demonstrated that morphine withdrawal, when paired with environmental context, leads to elevated corticosterone levels.^[^ ^18^ ^]^ Similarly, Elena et al. found that the acquisition of CPA was accompanied by increased corticosterone release in morphine‐withdrawn mice.^[^ ^19^ ^]^ These findings suggest that previously neutral cues, when associated with withdrawal, can acquire the capacity to elevate corticosterone upon re‐exposure. However, the role and mechanisms underlying it in the context‐triggered retrieval of morphine withdrawal memories (CTR‐MWM) remain poorly characterized.

In this study, we examined whether context could increase corticosterone levels in morphine‐withdrawal mice by measuring the serum corticosterone concentration using the Enzyme‐Linked Immunosorbent Assay (ELISA) technique, as well as whether this increase is involved in CTR‐MWM by investigating the impact of adrenalectomy (ADX) surgery‐induced suppression of corticosterone production on CPA. Then, we studied which kind of receptors of corticosterone in BLA neurons projecting to the nucleus accumbens core (BLA^→NAcC^ neurons) contributed to CTR‐MWM by examining the influence of inhibiting mineralocorticoid receptor (MR) or glucocorticoid receptor (GR), using pharmacological inhibitors, RNA silencing and immunofluorescence staining method, on CPA and associated c‐Fos expression of BLA^→NAcC^ neurons. Finally, we investigated how MR in BLA^→NAcC^ neurons activated BLA neurons to participate in CTR‐MWM using electrophysiological recording techniques.

## 2 Results

### 2.1 Corticosterone is Involved in Context‐Triggered Retrieval of Morphine Withdrawal Memories

**Figure 1:**
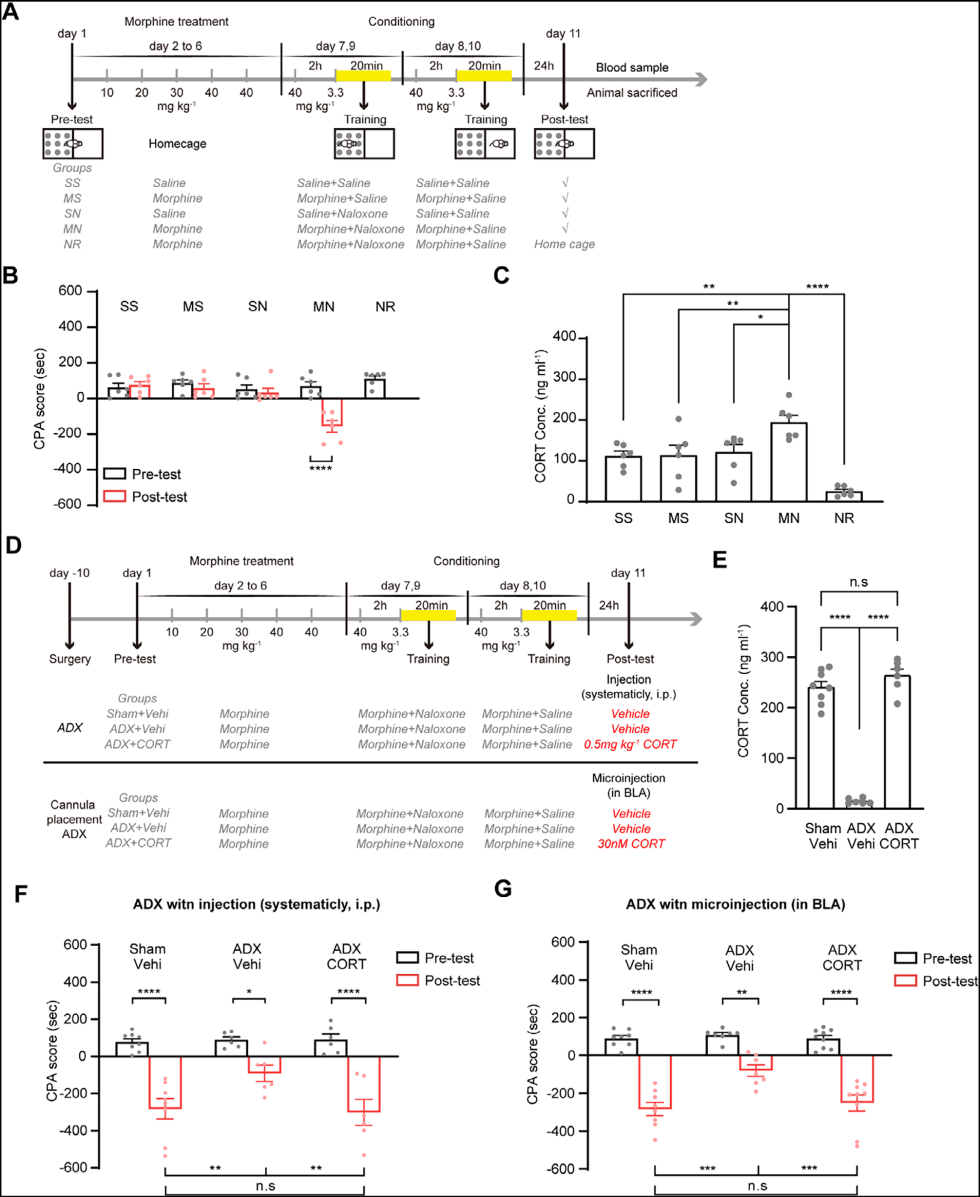
The role of corticosterone in CTR‐MWM. A). Experimental scheme. B). The average CPA scores in the SS, MS, SN, MN, and NR groups (*n* = 6 mice per group). C). The average concentrations of serum corticosterone in the five groups. D). Experimental scheme. E). The average concentrations of serum corticosterone in the Sham + Vehi (*n* = 8 mice), ADX + Vehi (*n* = 6 mice), and the ADX + CORT (*n* = 6 mice) groups. F). The average CPA scores in the three groups. G). The average CPA scores in the Sham + Vehi (*n* = 8 mice), ADX + Vehi (*n* = 7 mice) and the ADX + CORT (*n* = 9 mice) groups. ^*^ *P* < 0.05, ^**^ *P* < 0.01, ^***^ *P* < 0.001, ^****^ *P* < 0.0001. Means ± SEMs.

To investigate the role of corticosterone in CTR‐MWM, we first assessed whether the context could increase serum corticosterone levels in morphine‐withdrawal mice during CTR‐MWM using the ELISA technique. The mice were randomized into: Saline + Saline (SS) group, Morphine + Saline (MS) group, Saline + Naloxone (SN) group, Morphine + Naloxone (MN) group, Non‐retrieval (NR) group, and then underwent a CPA paradigm (except for NR group in which mice remained in their home cage without being through post‐test). As expected during the post‐test, mice in the MN group showed a pronounced aversion to the chamber associated with withdrawal; as a result, they spent reduced time in it and lead to an increased CPA score relative to the pre‐test; conversely, mice in the remaining groups exhibited no chamber aversion (Two‐way ANOVA, drug treatment factor, F _(3, 20)_ = 7.84, P < 0.01; test condition factor, F _(1, 20)_ = 21.50, P < 0.001; drug treatment × test condition, F _(3, 20)_ = 14.73, P < 0.0001; followed by Bonferroni's multiple comparisons test: pre‐ versus post‐test, P > 0.9999 in the SS, MS, SN group, P < 0.0001 in the MN group; Figure 1B). Serum samples were obtained from each of the four groups after the post‐test. An increase in serum corticosterone was observed in the MN group (194.38 ± 17.44 ng ml^−1^) compared with that in the SS group (112.55 ± 11.55 ng ml^−1^), MS group (113.78 ± 25.17 ng ml^−1^), SN group (121.94 ± 18.12 ng ml^−1^) and the NR group (25.33 ± 4.77 ng ml^−1^) (One‐way ANOVA, F _(4, 25)_ = 12.65, P < 0.0001; followed by Bonferroni's multiple comparisons test: MN versus SS, P < 0.01; MN versus MS, P < 0.01; MN versus SN, P < 0.05; MN versus NR, P < 0.0001; Figure 1C). This result indicates that the context associated with morphine withdrawal can elicit a significant increase in serum corticosterone levels.

We then examined the influence of inhibiting corticosterone production induced by ADX surgery on CTR‐MWM to study the role of context‐induced increase in serum corticosterone. Three groups were involved: the Sham + Vehi group, which consisted of mice that were subjected to ADX surgical procedure but with the adrenal gland intact and received a vehicle injection (intraperitoneal, i.p.) before CPA post‐test; the ADX + Vehi group, which consisted of mice that were subjected to ADX surgery and received a vehicle injection (i.p.) before CPA post‐test; the ADX + CORT group, which consisted of mice that were subjected to ADX surgery and received a 0.5 mg kg^−1^ corticosterone injection (i.p.) before CPA post‐test. Following ten days of recuperation, the mice underwent a behavioral paradigm, as shown in Figure 1D. Serum samples were collected after the post‐test to confirm the inhibitory impact of ADX on the corticosterone production. The results indicated that the concentration of serum corticosterone in the ADX + Vehi group (14.99 ± 2.17 ng ml^−1^) was significantly decreased compared with the Sham + Vehi group (240.14 ± 11.84 ng ml^−1^); administration of 0.5 mg kg^−1^ corticosterone to ADX mice before post‐test (263.32 ± 13.29 ng ml^−1^) could increase concentration of serum corticosterone to the level similar to that of the Sham + Vehi group (One‐way ANOVA, F _(2, 17)_ = 149.60, P < 0.0001; followed by Bonferroni's multiple comparisons test: Sham + Vehi versus ADX + Vehi, P < 0.0001; Sham + Vehi versus ADX + CORT, P = 0.4295; ADX + Vehi versus ADX + CORT, P < 0.0001; Figure 1E). This result proves that ADX surgery can inhibit corticosterone production in the mice of the ADX group. According to the behavior test results, the mice in the ADX + Vehi group exhibited decreased CPA scores compared to the Sham + Vehi group. Meanwhile, administration of corticosterone to ADX mice before the post‐test could restore the decreased CPA scores to levels comparable to the Sham + Vehi group (Two‐way ANOVA, treatment factor, F _(2, 17)_ = 3.30, P = 0.0615; test condition factor, F _(1, 17)_ = 81.21, P < 0.0001; treatment × test condition, F _(2, 17)_ = 3.44, P = 0.0557; followed by Bonferroni's multiple comparisons test: pre‐ versus post‐test, P < 0.0001 in the Sham + Vehi group, P < 0.05 in the ADX + Vehi group, P < 0.0001 in the ADX + CORT group; the post‐test of the Sham + Vehi group versus the ADX + Vehi group, P < 0.01, the Sham + Vehi group versus the ADX + CORT group, P > 0.05, the ADX + Vehi group versus the ADX + CORT group, P < 0.01; Figure 1F). This result suggests that corticosterone is involved in CTR‐MWM.

To further investigate the role of corticosterone in CTR‐MWM, we then studied the impact of BLA corticosterone intervention in ADX mice on CTR‐MWM. Mice that underwent cannula implantation in the BLA were randomized into: the Sham + Vehi group, consisted of mice that subjected to an ADX surgical procedure but with the adrenal gland intact and received a bilaterally BLA microinjection of vehicle before CPA post‐test; the ADX + Vehi group, which consisted of mice that were subjected to ADX surgery and received a bilaterally BLA microinjection of vehicle before CPA post‐test; the ADX + CORT group, consisted of mice that subjected to ADX surgery and received a bilaterally BLA microinjection of 30nM corticosterone before CPA post‐test. Following ten days of recuperation, the mice underwent a behavioral paradigm, as shown in Figure 1D. The results showed that the mice in the ADX + Vehi group exhibited decreased CPA scores compared to the Sham + Vehi group as expected, and local infusion of corticosterone into BLA before the post‐test similarly restored a significant CPA score in ADX mice (Two‐way ANOVA, treatment factor, F _(2, 21)_ = 8.48, P < 0.05; test condition factor, F _(1, 21)_ = 153.4, P < 0.0001; treatment × test condition, F _(2, 21)_ = 5.07, P = 0.0159; followed by Bonferroni's multiple comparisons test: pre‐ versus post‐test, P < 0.0001 in the Sham + Vehi group, P < 0.01 in the ADX + Vehi group, P < 0.0001 in the ADX + CORT group; the post‐test of the Sham + Vehi group versus the ADX + Vehi group, P = 0.0001, the Sham + Vehi group versus the ADX + CORT group, P > 0.9999, the ADX + Vehi group versus the ADX + CORT group, P < 0.001; Figure 1G). These results confirm that the context‐induced increase in serum corticosterone is involved in CTR‐MWM.

### 2.2 Corticosterone Participates in Context‐Triggered Retrieval of Morphine Withdrawal Memories Through Acting on MR, but not GR, in the BLA

**Figure 2:**
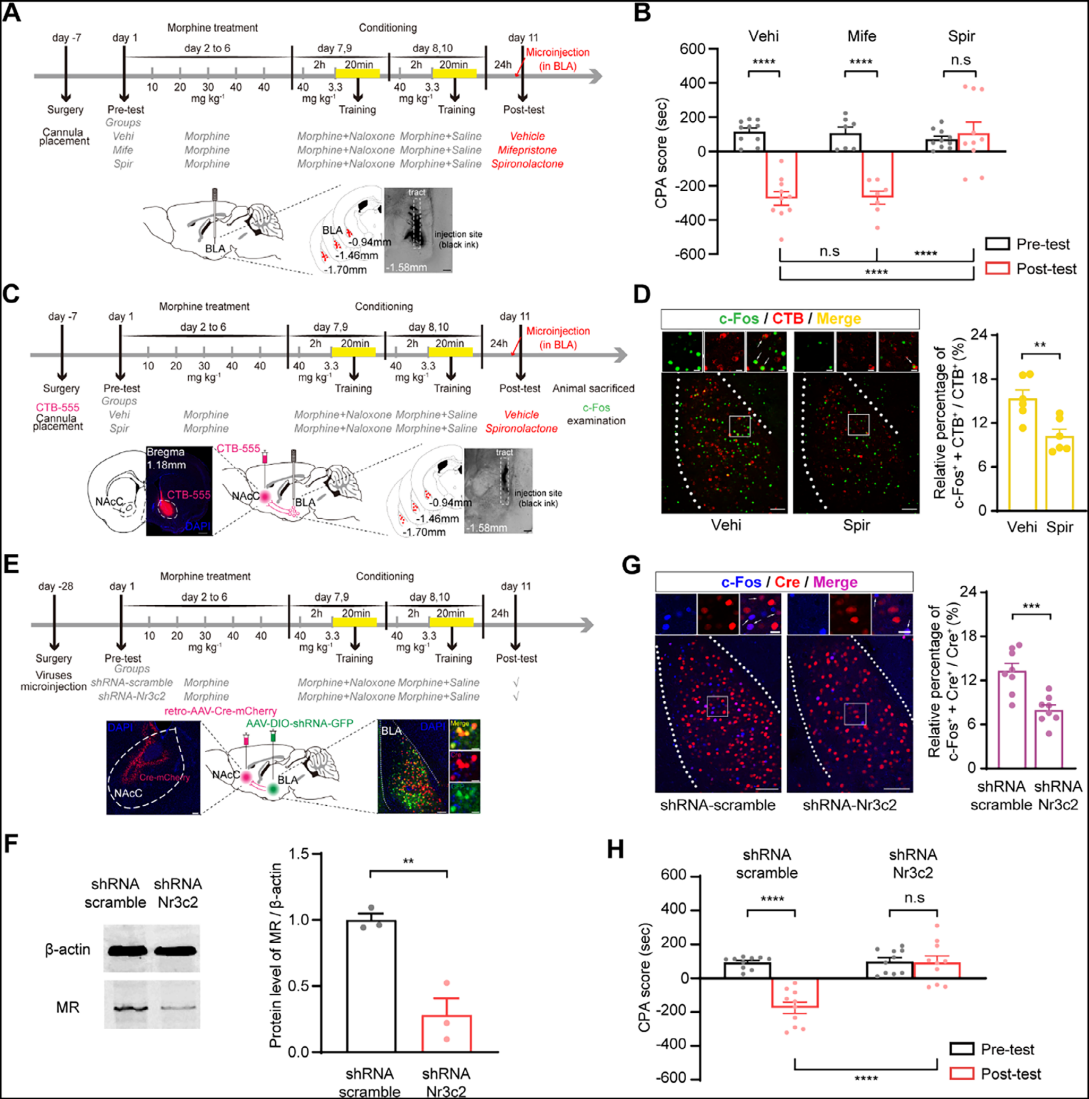
The role of MR in activation of BLA^→NAcC^ neurons during CTR‐MWM. A). Experimental scheme. B). The average CPA scores in the Vehi (*n* = 10 mice), Mife (*n* = 7 mice), and Spir (*n* = 10 mice) groups. C). Experimental scheme, diagram, and anatomical location of the microinjection sites in NAcC and cannula placement in BLA. Scale bars, 500 and 100 µm. D). Immunofluorescence analysis of c‐Fos^+^ (green) + CTB‐555^+^ (red) co‐labeled neurons in BLA. Magnified images show the boxed area. Scale bars, 100 and 20 µm (*n* = 6 mice per group). E). Experimental scheme, diagram of microinjection sites, and the expression of virus in BLA. Scale bars, 100 and 10 µm. F). The relative expression levels of MR in BLA from shRNA‐scramble and shRNA‐Nr3c2 mice (*n* = 3 samples from 9 mice). G). Immunofluorescence analysis of c‐Fos^+^ (blue) + Cre^+^ (red) co‐labeled neurons in BLA. Magnified images show the boxed area. Scale bars, 100 and 20 µm (n = 8 mice per group). H). The average CPA scores in the shRNA‐scramble and shRNA‐Nr3c2 groups (*n* = 10 mice per group). ^**^ *P* < 0.01, ^***^ *P* < 0.001, ^****^ *P* < 0.0001. Means ± SEMs.

There are two different subtypes of corticosterone receptors, the high‐affinity MR and the low‐affinity GR.^[^ ^20^, ^21^, ^22^, ^23^ ^]^ Brain areas like the BLA, which express both MR and GR,^[^ ^21^, ^23^ ^]^ have been shown to contribute to CTR‐MWM.^[^ ^11^, ^24^ ^]^ To study which subtype of corticosterone receptor in the BLA mediates CTR‐MWM, first, we examined the impact of inhibiting either GR or MR in the BLA, using the GR inhibitor mifepristone^[^ ^25^ ^]^ or the MR inhibitor spironolactone,^[^ ^26^ ^]^ on CTR‐MWM. Following a week of recuperation after BLA cannula implantation surgery, the mice underwent a behavioral paradigm, as shown in **Figure** **2**A where they were randomized into three groups according to the microinjection they received before the post‐test: vehicle (the Vehi group), mifepristone (the Mife group), and spironolactone (the Spir group) separately. The results indicated that a pronounced aversion to the chamber associated with withdrawal was triggered by the context in both the Vehi and Mife groups, but not in the Spir group (Two‐way ANOVA, drug treatment factor, F _(2, 24)_ = 13.15, P = 0.0001; test condition factor, F _(1, 24)_ = 52.08, P < 0.0001; drug treatment x test condition, F _(2, 24)_ = 18.16, P < 0.0001; followed by Bonferroni's multiple comparisons test: pre‐ versus post‐test, P < 0.0001 in the Vehi group, P < 0.0001 in the Mife group and P > 0.9999 in the Spir group; the post‐test of Vehi group versus Mife group, P > 0.9999, the Vehi group versus Spir group, P < 0.0001, the Mife group versus Spir group, P < 0.0001; Figure 2B). This result suggests that the effect of corticosterone on CTR‐MWM is mediated by MR, but not by GR, in the BLA.

### 2.3 MR in BLA^→NAcC^ Neurons Mediates Context‐Triggered Retrieval of Morphine Withdrawal Memories

Our prior research indicated that BLA^→NAcC^ neurons participated in CTR‐MWM.^[^ ^27^ ^]^ In order to verify this claim, we reexamined whether context was able to activate BLA^→NAcC^ neurons through examination of the expression of c‐Fos (a marker of neuronal activation) in BLA^→NAcC^ neurons labeled with retrograde tracer CTB‐555 bilaterally injected in NAcC. Following a week of recuperation, as shown in Figure S1A (Supporting Information), the mice were then randomized into SS, MS, SN, and MN groups and underwent a behavioral paradigm. The result indicated that mice in the MN group showed a pronounced aversion to the chamber associated with withdrawal, while in other three groups, mice exhibited no aversion to either chamber (Two‐way ANOVA, drug treatment factor, F _(3, 20)_ = 14.43, P < 0.0001; test condition factor, F _(1, 20)_ = 6.57, P < 0.05; drug treatment × test condition, F _(3, 20)_ = 22.96, P < 0.0001; Figure S1B, Supporting Information). 90 min following post‐test, mice in four groups were euthanized, and immunofluorescence staining was employed to access the contextual effect on c‐Fos expression in BLA. Results indicated that in the MN group, the average proportion of c‐Fos^+^ + CTB‐555^+^ neurons in CTB‐555^+^ neurons in the BLA was significantly elevated (14.32 ± 0.41%) compared to the SS (5.77 ± 0.21%), MS (5.15 ± 0.38%), and SN (6.44 ± 0.39%) groups (One‐way ANOVA, F _(3, 20)_ = 144.00, P < 0.0001; followed by Bonferroni's multiple comparisons test: MN versus SS, P < 0.0001; MN versus MS, P < 0.0001; MN versus SN, P < 0.0001; Figure S1C,D, Supporting Information). This data indicates that re‐exposure to context could activate BLA^→NAcC^ neurons in morphine‐withdrawal mice. Subsequently, we analyzed the impact of chemogenetic inactivation of BLA^→NAcC^ neurons on CPA in morphine‐withdrawal mice to investigate the role of BLA^→NAcC^ neurons in CTR‐MWM. AAV‐mCherry‐WGA‐Cre was microinjected bilaterally into the NAcC, and meanwhile AAV‐DIO‐hM4D(i)‐EGFP or AAV‐DIO‐EGFP was microinjected bilaterally into the BLA. Two groups of hM4D(i)‐expressing mice were involved: the hM4Di + Sal group, which consisted of mice received an injection of vehicle (i.p.) prior to post‐test; the hM4Di + CNO group, consisted of mice received an injection of clozapine‐n‐oxide (CNO), a substance that inhibits the activity of BLA^→NAcC^ neurons, simultaneously. As empty controls, mice that expressed only EGFP without hM4Di (EGFP + CNO group) were given an injection of CNO simultaneously. Following four weeks of recuperation after surgery, the mice underwent a behavioral paradigm, as shown in Figure S1E (Supporting Information). The data indicated that a pronounced aversion to the chamber associated with withdrawal was triggered by the context in both the hM4Di + Sal and EGFP + CNO groups. However, this aversion was not observed in the hM4Di + CNO group (Two‐way ANOVA, drug treatment factor, F _(2, 25)_ = 7.01, P < 0.01; test condition factor, F _(1, 25)_ = 51.17, P < 0.0001; drug treatment × test condition, F _(2, 25)_ = 6.69, P < 0.01; followed by Bonferroni's multiple comparisons test: pre‐ versus post‐test, P < 0.0001 in the hM4Di + Sal group, P < 0.0001 in the EGFP + CNO group and P = 0.6636 in the hM4Di + CNO group; the post‐test of hM4Di + Sal group versus EGFP + CNO group, P > 0.9999, the hM4Di + Sal group versus hM4Di + CNO group, P = 0.0002, the EGFP + CNO group versus hM4Di + CNO group, P < 0.0001; Figure S1F, Supporting Information). These findings imply that the activation of BLA^→NAcC^ neurons is essential for CTR‐MWM.

On this basis, we further studied whether MR mediated context‐induced activation of BLA^→NAcC^ neurons during CTR‐MWM by detecting the impact of inhibiting MR, using the MR inhibitor spironolactone, on context‐induced activation of BLA^→NAcC^ neurons. For retrograde labeling, CTB‐555 was injected bilaterally into the NAcC before cannula placement in the BLA. After the post‐test of the CPA paradigm illustrated in Figure 2C, the mice in the Vehi and Spir groups were euthanized, and then c‐Fos expression in BLA^→NAcC^ neurons was accessed. The average proportion of c‐Fos^+^ + CTB‐555^+^ neurons in CTB‐555^+^ neurons in the Spir group was 10.24 ± 0.92%, and this was much lower than that in the Vehi group (15.35 ± 1.16%) (Student's t test, t = 3.3447, P < 0.01; Figure 2D). This result suggests that MR in BLA is involved in the activation of BLA^→NAcC^ neurons during CTR‐MWM.

Furthermore, we investigated the role of MR in BLA^→NAcC^ neurons in CTR‐MWM by detecting the impact of suppressed MR expression in these neurons. We used a retro‐AAV‐Cre‐mCherry virus and a recombinant AAV virus that could express a short hairpin RNA (shRNA) targeting Nr3c2 in a Cre‐dependent manner. This allowed us to specifically silence MR expression in BLA^→NAcC^ neurons and study its impact on CTR‐MWM. Mice were randomized into: the shRNA‐scramble group, bilaterally microinjected with Retro‐AAV‐Cre‐mCherry into NAcC and Cre‐dependent AAV‐DIO‐shRNA (scramble) into BLA; the shRNA‐Nr3c2 group, bilaterally microinjected with Retro‐AAV‐Cre‐mCherry into NAcC and Cre‐dependent AAV‐DIO‐shRNA (Nr3c2) into BLA. Four weeks after injection, the expression of Retro‐AAV‐Cre‐mCherry in NAcC and AAV‐DIO‐shRNA (scramble/Nr3c2) in the BLA could be detected (Figure 2E). The decreased expression of MR in the BLA was confirmed by western blotting (Student's t test, t = 5.3070, P < 0.01; Figure 2F) and in the BLA^→NAcC^ neurons by immunofluorescence staining separately (Student's t test, t = 10.5300, P < 0.0001; Figure S2, Supporting Information). After the behavioral paradigm shown in Figure 2E, the mice from both two groups were euthanized 90 min following the post‐test. Then the c‐Fos expression in BLA^→NAcC^ neurons was detected, revealing that in the shRNA‐Nr3c2 group, the average proportion of c‐Fos^+^ + Cre^+^ neurons in Cre^+^ neurons in the BLA was significantly reduced (7.98 ± 0.67%) compared to the shRNA‐scramble group (13.30 ± 1.04%) (Student's t test, t = 4.2860, P < 0.001; Figure 2G). The CPA result indicated that a pronounced aversion to the chamber associated with withdrawal was triggered by the context in the shRNA‐scramble group, but not in the shRNA‐Nr3c2 group (Two‐way ANOVA, treatment factor, F _(1, 18)_ = 16.29, P < 0.001; test condition factor, F _(1, 18)_ = 40.30, P < 0.0001; drug treatment × test condition, F _(1, 18)_ = 36.32, P < 0.0001; followed by Bonferroni's multiple comparisons test: pre‐ versus post‐test, P < 0.0001 in the shRNA‐scramble group, P > 0.9999 in the shRNA‐Nr3c2 group; the post‐test of shRNA‐scramble group versus shRNA‐Nr3c2 group, P < 0.0001); Figure 2H). These results suggest that corticosterone activates BLA^→NAcC^ neurons via MR during CTR‐MWM and that MR in BLA^→NAcC^ neurons mediates CTR‐MWM.

### 2.4 MR Increases Presynaptic Glutamate Release, Participates in D1 receptor‐induced Increase in Presynaptic Glutamate Release, and Postsynaptic AMPA Currents of BLA^→NAcC^ Neurons during Context‐triggered Retrieval of Morphine Withdrawal Memories

**Figure 3:**
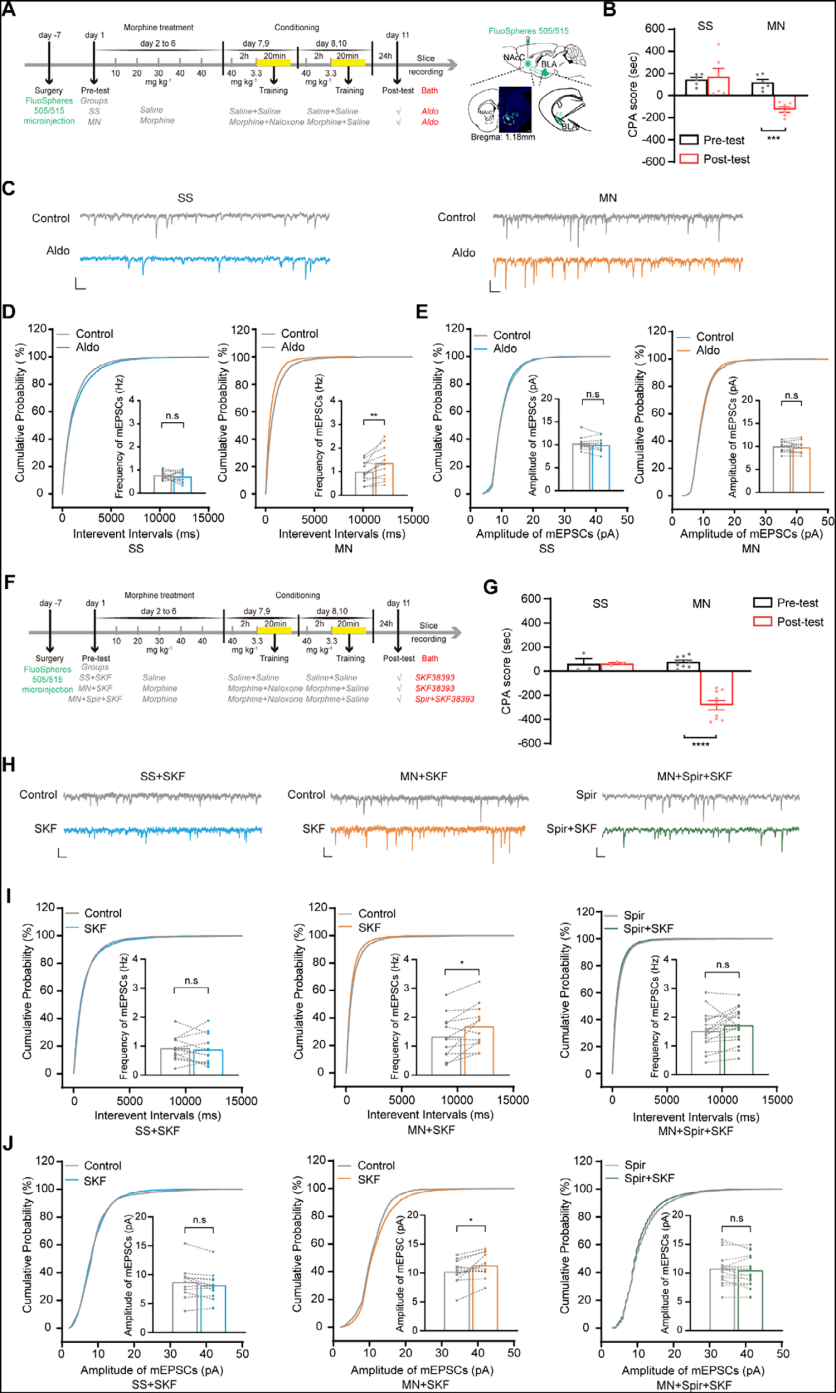
The influence of MR activation on the mEPSCs of BLA^→NAcC^ neurons and the role of MR in D1 receptors' effect on mEPSCs of BLA^→NAcC^ neurons during CTR‐MWM. A). Experimental scheme, diagram of the microinjection sites in NAcC. Scale bars, 500 µm. B). The average CPA scores in the SS and MN groups (*n* = 6 mice per group). C). Representative traces of mEPSCs before (Control) and after 10nM‐Aldo treatment in BLA^→NAcC^ neuron. Scale bars, 5 pA, 200 ms. D). Probability cumulative curves and graphs of mEPSC frequency before (Control) and after 10nm‐Aldo treatment in the SS group (*n* = 13 cells from 6 mice) and the MN group (*n* = 13 cells from 6 mice). E).Probability cumulative curves and graphs of mEPSC amplitude before (Control) and after 10nm‐Aldo treatment in the SS group and the MN group. F). Experimental scheme. G). The average CPA scores in the SS (*n* = 3 mice) and MN (*n* = 9 mice) groups. H). Representative traces of mEPSCs before and after drug treatment in BLA^→NAcC^ neurons. Scale bars, 5 pA, 200 ms. I).Probability cumulative curves and graphs of mEPSC frequency before (Control) and after drug treatment in the SS + SKF group (left, *n* = 12 cells from 3 mice), the MN + SKF group (middle, *n* = 12 cells from 3 mice) and the MN + Spir + SKF group (right, *n* = 17 cells from 6 mice). J).Probability cumulative curves and graphs of mEPSC amplitude before (Control) and after drug treatment in the SS + SKF group, the MN + SKF group, and the MN + Spir + SKF group. ^*^ *P* < 0.05, ^**^ *P* < 0.01, ^***^ *P* < 0.001. Means ± SEMs.

To study how MR activated BLA^→NAcC^ neurons during CTR‐MWM, we first investigated the influence of the MR agonist aldosterone^[^ ^26^ ^]^ on glutamatergic input of BLA^→NAcC^ neurons using the frequency of miniature excitatory postsynaptic currents (mEPSCs) as an index of presynaptic glutamate release and the amplitude of mEPSCs as an index of the sensitivity of postsynaptic AMPA (α‐Amino‐3‐hydroxy‐5‐methyl‐4‐isoxazolepropionic Acid) receptors to glutamate.^[^ ^28^, ^29^ ^]^ For retrograde labeling, fluorescent microsphere was injected bilaterally into the NAcC. Following a week of recuperation, the mice were randomized into the SS and MN groups and underwent a behavioral paradigm, as shown in **Figure** **3**A. The CPA result indicated that in MN group, a pronounced aversion to the chamber associated with withdrawal was triggered by the context, but not in SS group (Two‐way ANOVA, drug treatment factor, F _(1, 10)_ = 10.11, P < 0.01; test condition factor, F _(1, 10)_ = 10.81, P < 0.01; drug treatment × test condition, F _(1, 10)_ = 17.02, P < 0.01; Figure 3B). Mice in both groups were euthanized after the post‐test of the behavioral paradigm, and brain slices containing BLA were obtained. Figure 3C illustrates typical raw current traces recorded in microsphere‐labeled neurons before and after 10 nm Aldo in the two groups. Application of 10 nm Aldo had no significant effect on the average mEPSCs frequency in the SS group, but increased the average mEPSCs frequency in the MN group (from 0.77 ± 0.05 Hz to 0.71 ± 0.07 Hz in SS group, paired *t* test, t = 0.7624, P = 0.4605; from 1.00 ± 0.12 Hz to 1.36 ± 0.18 Hz in MN group, paired *t* test, t = 3.7690, P < 0.01; Figure 3D). Application of 10 nm Aldo had no influence on the average mEPSCs amplitude in both two groups (from 10.26 ± 0.36 pA to 9.93 ± 0.39 pA in SS group, paired t test, t = 1.5860, P = 0.1388; from 10.00 ± 0.31 pA to 9.79 ± 0.32 pA in MN group, paired t test, t = 1.1120, P = 0.2880; Figure 3E). This data indicates that the MR‐evoked activation of BLA^→NAcC^ neurons is related to an increase in presynaptic glutamate release, rather than sensitivity of postsynaptic AMPA receptors to glutamatergic input of BLA^→NAcC^ neurons.

**Figure 4:**
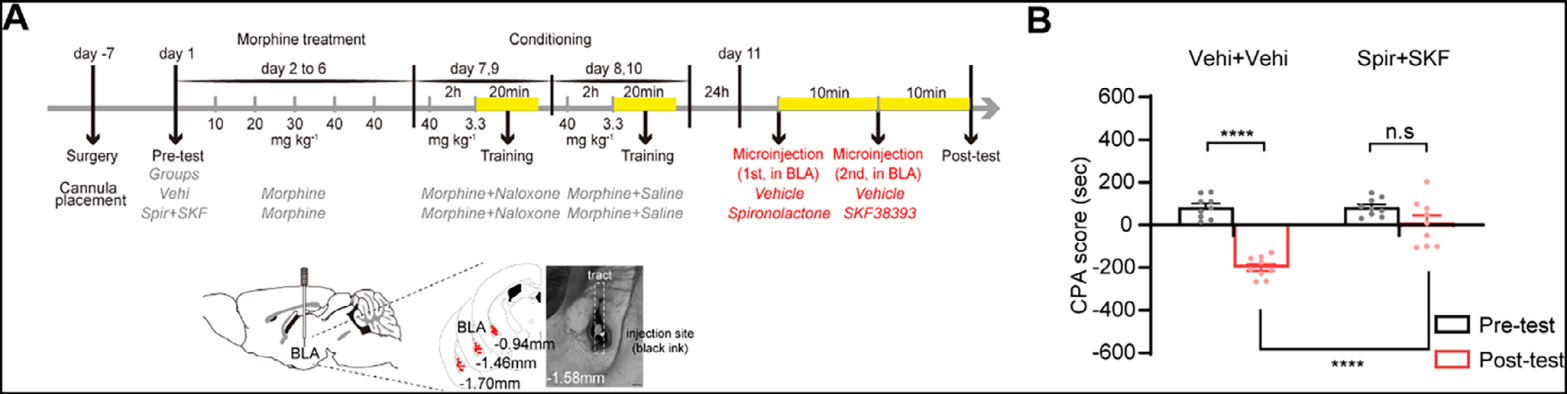
The effect of D1R activation in BLA on MR blockade during CTR‐MWM. A). Experimental scheme, diagram, and anatomical location of cannula placement in BLA. Scale bars, 500 µm. B). The average CPA scores in the Vehi + Vehi and Spir + SKF groups (*n* = 9 mice each group). ^****^ *P* < 0.0001. Means ± SEMs.

Previous studies of our lab found that the activation of dopamine D1 receptors in BLA^→NAcC^ neurons could increase the presynaptic glutamate release and sensitivity of postsynaptic AMPA receptors to glutamatergic input of BLA^→NAcC^ neurons.^[^ ^27^ ^]^ Here, we examined whether MR participated in this D1 receptor‐induced increase in the pre‐ and post‐synaptic changes of BLA^→NAcC^ neurons during CTR‐MWM. In order to retrogradely label BLA^→NAcC^ neurons, fluorescent microsphere was injected bilaterally into the NAcC. Following a week of recuperation, the mice were first randomized into the SS and MN groups. After behavioral paradigm as shown in Figure 3F, the CPA result indicated that a pronounced aversion to the chamber associated with withdrawal was triggered by the context in the MN groups, but not in the SS group (Two‐way ANOVA, drug treatment factor, F _(1, 10)_ = 17.41, P < 0.01; test condition factor, F _(1, 10)_ = 20.95, P = 0.0010; drug treatment × test condition, F _(1, 10)_ = 21.60, P < 0.001; Figure 3G). Then, the brain slice containing BLA from these mice was collected. In the SS group, D1 receptor agonist SKF38393 (10 µm) was added to the perfusate after the control baseline was recorded (SS + SKF group). Mice in the MN group were further divided into two subgroups: in MN + SKF group, SKF 38393 (10 µm) was added to the perfusate after the control baseline was recorded; in MN + Spir + SKF group, MR antagonist spironolactone (1 µm) was first added to the perfusate when the control baseline was stable and SKF38393 (10 µm) was added after the control baseline was recorded. Typical raw current traces in microsphere‐labeled neurons before and after drug treatment in all groups are shown in Figure 3H. Application of SKF38393 had no effect on the average mEPSCs frequency in the SS + SKF group (from 0.93 ± 0.12 Hz to 0.89 ± 0.15 Hz, paired *t* test, t = 0.4128, P = 0.6877), but significantly increased that in the MN + SKF group (from 1.33 ± 0.22 Hz to 1.69 ± 0.22 Hz, paired *t* test, t = 2.9580, P < 0.05). Furthermore, this enhanced effect of SKF38393 was canceled in the MN + Spir + SKF group (from 1.52 ± 0.15 Hz to 1.71 ± 0.15 Hz, paired *t* test, t = 1.8980, P = 0.0739; Figure 3I). Similarly, application of SKF38393 had no discernible influence on the mEPSCs amplitude in the SS + SKF group (from 8.71 ± 0.81 pA to 8.15 ± 0.70 pA, paired *t* test, t = 1.974, P = 0.0659), but significantly increased that in the MN + SKF group (from 10.22 ± 0.60 pA to 11.30 ± 0.60 pA, paired *t* test, t = 2.5980, P < 0.05). This enhanced effect of SKF38393 was also canceled in the MN + Spir + SKF group (from 10.80 ± 0.65 pA to 10.50 ± 0.64 pA, paired *t* test, t = 1.314, P = 0.2074; Figure 3J). These results imply that MR participates in the D1 receptor‐induced increase in the presynaptic glutamate release and sensitivity of postsynaptic AMPA receptors to glutamatergic input of BLA^→NAcC^ neurons. To functionally validate this interaction in vivo, we performed a behavioral experiment testing whether D1R activation overrides MR blockade during CPA retrieval. Mice undergoing cannula implantation in the BLA were randomized into two groups: Vehi + Vehi group: received vehicle microinjections 20 and 10 min prior to the post‐test; Spir + SKF group: received spironolactone microinjection 20 min before and SKF38393 microinjection 10 min before the post‐test (**Figure** **4**A). The result showed that context exposure triggered pronounced aversion to the withdrawal‐associated chamber in the Vehi + Vehi group, but not in the Spir + SKF group (Two‐way ANOVA, treatment factor, F _(1,16)_ = 18.92, P < 0.001; test condition factor, F _(1,16)_ = 70.51, P < 0.0001; drug treatment × test condition, F _(1,16)_ = 24.15, P < 0.001; followed by Bonferroni's multiple comparisons test: pre‐ versus post‐test, P < 0.0001 in the Vehi + Vehi group, P = 0.0511 in the Spir + SKF group; the post‐test of Vehi + Vehi group versus Spir + SKF group, P < 0.0001); Figure 4B). This result indicated that D1R activation in the BLA failed to override MR blockade during CPA retrieval.

During mEPSCs recording, tetrodotoxin (TTX, 1 µm), N‐Methyl‐D‐Aspartate receptor agonist APV (50 µm), and Gamma‐Aminobutyric Acid Type A receptor antagonist SR95531 (10 µm) were used to isolate AMPA‐mediated currents. However, kainate receptors (KARs) have been reported to be capable of modulating AMPA receptor‐mediated synaptic transmission.^[^ ^30^, ^31^ ^]^ To exclude the influence of KARs on the MR or D1R's effect on the mEPSCs in BLA^→NAcC^ neurons, we reexamined the effect of 10 nm Aldo and SKF38393 on the frequency and amplitude of mEPSCs of BLA^→NAcC^ neurons in morphine‐withdrawal mice. KAR antagonist UBP310 (30 µm)^[^ ^32^, ^33^ ^]^ was added into the perfusate (Figure S3A, Supporting Information). The results showed that 10 nm Aldo could still increase the average mEPSCs frequency of BLA^→NAcC^ neurons (from 0.81 ± 0.11 Hz to 1.22 ± 0.15 Hz, paired t test, t = 4.7320, P < 0.001; Figure S3C, Supporting Information). Also, SKF38393 increased the average mEPSCs frequency of BLA^→NAcC^ neurons (from 0.65 ± 0.11 Hz to 1.06 ± 0.16 Hz, paired *t* test, t = 4.5460, P < 0.001) and average mEPSCs amplitude of BLA^→NAcC^ neurons (from 8.17 ± 0.35 pA to 9.25 ± 0.45 pA, paired *t* test, t = 3.5290, P < 0.01; Figure S3D, Supporting Information). This result suggests that MR exerts the above effect in a KAR‐independent manner during CTR‐MWM.

### 2.5 MR Increases Intrinsic Excitability of BLA^→NAcC^ Neurons During Context‐Triggered Retrieval of Morphine Withdrawal Memories

**Figure 5:**
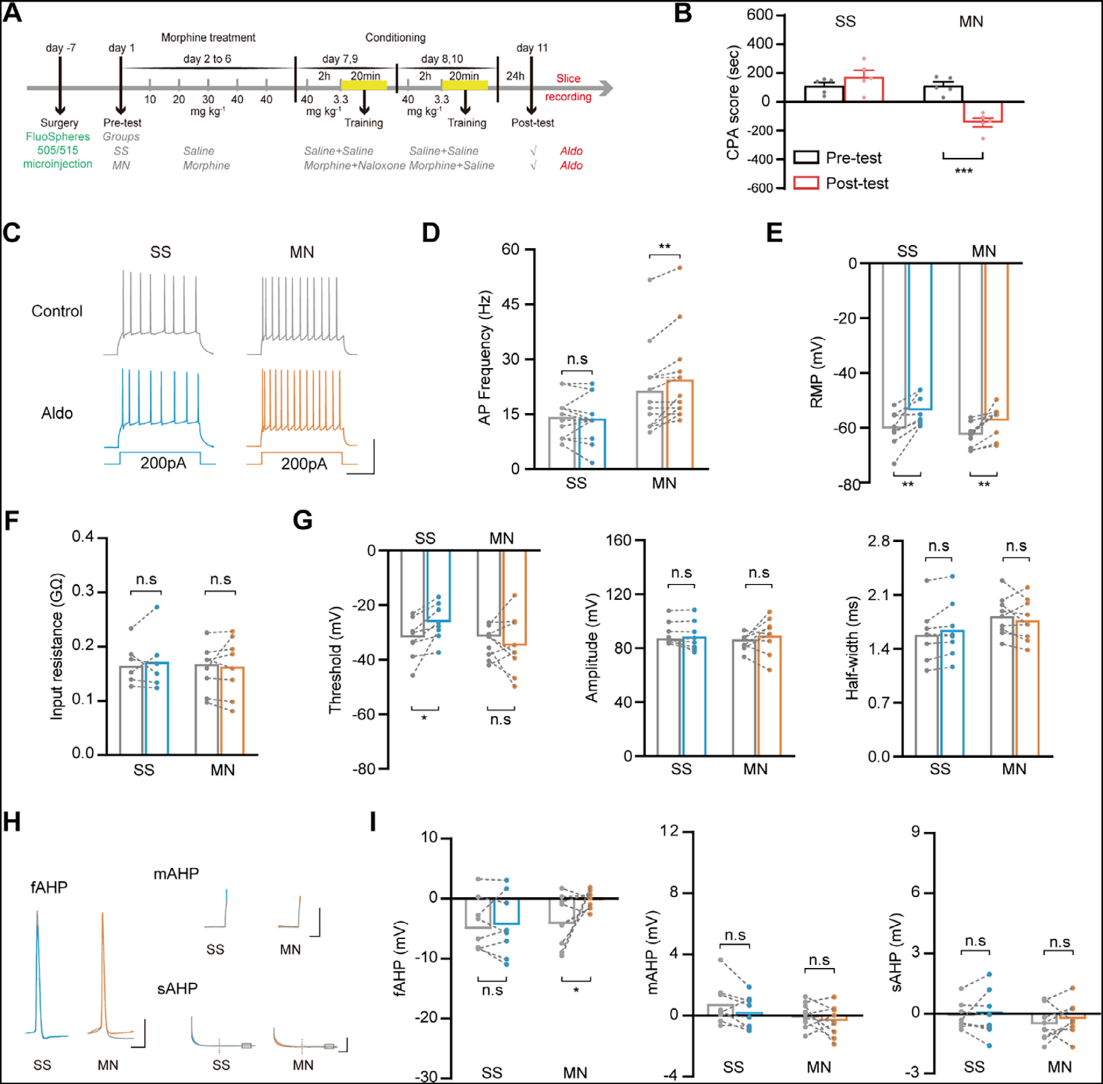
The influence of MR activation on the intrinsic excitability of BLA^→NAcC^ neurons during CTR‐MWM. A). Experimental scheme. B). The average CPA scores in the SS and MN groups (*n* = 5 mice per group). C). Typical AP traces in response to 200pA depolarizing currents before (Control) and after 10nm‐Aldo treatment in the SS and MN groups. Scale bars, 50 mV, 200 ms. D). The graphs of AP frequency before (Control) and after 10nM‐Aldo treatment in the SS (*n* = 13 cells from 5 mice) and MN (*n* = 13 cells from 5 mice) groups. E–G). The graphs of RMP, input resistance, threshold, amplitude, and half‐width of AP before (Control) and after 10nm‐Aldo treatment in the SS (*n* = 8 cells from 5 mice) and MN (*n* = 9 cells from 5 mice) groups. H). Typical AHP traces before (Control) and after 10nM‐Aldo treatment in two groups. Scale bars, 20 mV and 10 ms (fAHP), 20 mV and 50 ms (mAHP and sAHP). I). Average amplitude of fAHP (*left*), mAHP (*middle*), sAHP (*right*) before (Control) and after 10nm‐Aldo treatment in two groups. ^*^ *P* < 0.05, ^**^ *P* < 0.01, ^***^ *P* < 0.001. Means ± SEMs.

We also studied whether the activation of MR could increase the intrinsic excitability of BLA^→NAcC^ neurons by examining the change in the frequency of depolarizing current steps‐induced action potential (AP) firing in BLA^→NAcC^ neurons after the application of the MR agonist Aldo in the SS or MN group. In order to retrogradely label BLA^→NAcC^ neurons, fluorescent microsphere was injected bilaterally into the NAcC. Following a week of recuperation, mice were randomized into the SS and MN groups and then underwent a behavioral paradigm, as shown in **Figure** **5**A. The CPA result indicated that in the MN group, a pronounced aversion to the chamber associated with withdrawal was triggered by the context, but not in the SS group (Two‐way ANOVA, drug treatment factor, F _(1, 8)_ = 19.71, P < 0.01; test condition factor, F _(1, 8)_ = 10.87, P < 0.05; drug treatment × test condition, F _(1, 8)_ = 29.89, P < 0.001; Figure 5B). All mice were then euthanized, and brain slices containing BLA were obtained. Typical AP traces recorded in microsphere‐labeled neurons before and after 10 nm Aldo treatment in the two groups are shown in Figure 5C. Application of 10 nm Aldo had no discernible influence on the AP frequency in the SS group (from 14.23 ± 1.52 Hz to 13.85 ± 1.69 Hz), but significantly increased that in the MN group (from 21.41 ± 3.23 Hz to 24.49 ± 3.35 Hz; Two‐way ANOVA, treatment factor, F _(1, 24)_ = 6.07, P < 0.05; drug factor, F _(1, 24)_ = 5.82, P < 0.05; treatment × drug, F _(1, 24)_ = 9.61, P < 0.005; followed by Bonferroni's multiple comparisons test: the control versus the 10nM Aldo, P > 0.9999 in the SS group and P < 0.01 in the MN group; Figure 5D). These data indicate that MR increases the intrinsic excitability of BLA^→NAcC^ neurons in CTR‐MWM.

We then analyzed Aldo's effects on different characteristics of APs, including: resting membrane potential (RMP), input resistance (Rin), threshold, AP amplitude, half‐width, fast AHP (fAHP), medium AHP (mAHP), slow AHP (sAHP) to explore the underlying mechanisms by which MR activation induced an increase of the AP frequency of BLA^→NAcC^ neurons in morphine‐withdrawal mice. Application of 10 nM Aldo could decrease the RMP of BLA^→NAcC^ neurons both in the SS (from −60.34 ± 2.37 mV to −53.70 ± 1.96 mV) and MN (from −63.07 ± 1.48 mV to −57.42 ± 1.92 mV) group (Two‐way ANOVA, treatment factor, F _(1, 15)_ = 1.63, P = 0.2215; drug factor, F _(1, 15)_ = 33.97, P < 0.0001; treatment × drug, F _(1, 15)_ = 0.22, P = 0.6455; followed by Bonferroni's multiple comparisons test: the control versus the 10nm Aldo, P < 0.01 in the SS group and P < 0.01 in the MN group; Figure 5E). Application of 10 nm Aldo could decrease the threshold of BLA^→NAcC^ neurons in the SS group (from −32.37 ± 2.64 mV to −26.22 ± 2.37 mV) but had no influence on that in the MN group (from ‐33.76 ± 1.86 mV to ‐34.90 ± 3.52 mV) (Two‐way ANOVA, treatment factor, F _(1, 15)_ = 2.12, P = 0.1658; drug factor, F _(1, 15)_ = 2.40, P = 0.1425; treatment × drug, F _(1, 15)_ = 5.09, P < 0.05; followed by Bonferroni's multiple comparisons test: the control versus the 10nm Aldo, P < 0.05 in the SS group and P < 0.9999 in the MN group; Figure 5G). Rin, threshold, amplitude, and half‐width of AP remained unchanged both in the SS and MN groups (Figure 5F,G). For fAHP, mAHP and sAHP, which contribute differently to neuronal excitability based on their temporal dynamics and underlying currents,^[^ ^34^ ^]^ application of 10 nM Aldo in the SS group had no discernible influence on the amplitude of fAHP (from −4.27 ± 1.55 mV to −4.39 ± 1.85 mV), but significantly decreased that in the MN group (−3.77 ± 1.39 mV to −0.24 ± 0.51 mV; Two‐way ANOVA, treatment factor, F _(1, 15)_ = 1.85, P = 0.1936; drug factor, F _(1, 15)_ = 3.23, P = 0.0924; treatment × drug, F _(1, 15)_ = 3.69, P = 0.0738; followed by Bonferroni's multiple comparisons test: the control versus 10nm‐Aldo, P > 0.9999 in the SS group and P < 0.05 in the MN group; Figure 5I). For mAHP and sAHP, no significant effect on amplitude was observed with 10 nM Aldo in either the SS or MN group. These results indicate that the activation of MR may increase the intrinsic excitability of BLA^→NAcC^ neurons via inhibition of fAHP during CTR‐MWM.

## 3 Discussion

The primary conclusions of the current study are that corticosterone is involved in CTR‐MWM; corticosterone participates in CTR‐MWM through acting on MR, but not GR, in the BLA; MR in BLA^→NAcC^ neurons mediates CTR‐MWM; MR increases presynaptic glutamate release, and meanwhile, participates in D1 receptor‐induced increases in presynaptic glutamate release and postsynaptic AMPA currents; MR increases intrinsic excitability of BLA^→NAcC^ neurons during CTR‐MWM. These findings suggest that peripheral corticosterone, traditionally viewed as a stress hormone, also plays a role in CTR‐MWM by modulating limbic circuit activity via MR signaling.

While most prior studies have emphasized central neural circuits in CTR‐DWM, our findings support and extend earlier work^[^ ^12^, ^35^, ^36^, ^37^, ^38^, ^39^, ^40^ ^]^ by showing that peripheral corticosterone also participates in CTR‐MWM. However, how context elicits an increase in corticosterone in morphine‐withdrawal mice remains to be investigated. We speculate that when drug withdrawal symptoms which are associated with an increase in serum corticosterone, occur, in addition to being associated with drug withdrawal symptoms, the environmental context may also establish an association with corticosterone via neural circuits that link to the HPA axis, thus stimulating the biosynthesis and secretion of corticosterone.^[^ ^41^, ^42^ ^]^ Therefore, the originally neutral context acquires the ability to induce an increase in serum corticosterone. The possible neural circuits may include projections from the hippocampus, amygdala, bed nucleus of the stria terminalis, PVT, and prefrontal cortex to the paraventricular nucleus neurons, because these circuits are related to context and the HPA axis, but it remains to be investigated.^[^ ^43^, ^44^, ^45^ ^]^

In our experiments, ADX significantly reduced serum corticosterone levels and attenuated CPA, suggesting that corticosterone was involved in CTR‐MWM. However, some residual aversive behavior was observed in ADX mice, indicating that, in addition to corticosterone, CPA may be sustained by alternative compensatory mechanisms or parallel pathways such as non‐corticosterone stress‐related signals.

Serum corticosterone can penetrate the brain swiftly and bind to corticosterone receptors. There are two distinct subtypes of corticosterone receptors, the high‐affinity MR and the low‐affinity GR. GR is widely expressed across the brain, while MR is predominantly localized to limbic areas, including the prefrontal cortex, amygdala, and hippocampus.^[^ ^21^ ^]^ Since the BLA is a crucial brain region mediating CTR‐MWM,^[^ ^11^, ^12^ ^]^ here, we investigated the role of GR and MR of the BLA in CTR‐MWM. Our result shows that MR, rather than GR, in the BLA is involved in CTR‐MWM. This result aligns with Rimmele et al.'s finding that blocking MR impaired memory retrieval.^[^ ^46^ ^]^

Among the various projection neurons of the BLA, our prior work found that BLA^→NAcC^ neurons and prelimbic cortex‐projecting BLA (BLA^→PrL^) neurons participate in CTR‐MWM.^[^ ^27^ ^]^ In the present study, we focused on the BLA^→NAcC^ pathway and found that corticosterone activates BLA^→NAcC^ neurons via MR to contribute to CTR‐MWM. However, we acknowledge that our findings do not rule out the possibility that corticosterone also acts on BLA^→PrL^ neurons. This remains an important avenue for future investigation.

Mechanistically, we show that activation of MR could increase presynaptic glutamate release to the BLA^→NAcC^ neurons, which aligns with previous findings that MR could increase the excitability of neurons in the hippocampus and amygdala.^[^ ^47^, ^48^, ^49^, ^50^ ^]^ Moreover, our work shows that MR can activate BLA^→NAcC^ neurons via two postsynaptic pathways: one is to participate in D1 receptor‐induced increases in postsynaptic AMPA currents, and another is to increase the intrinsic excitability of BLA^→NAcC^ neurons during CTR‐MWM. This indicates a crosstalk between the corticosterone system and dopamine system in BLA during CTR‐MWM.

There are two kinds of MRs: one is genomic MR (gMRs) that exist in the cytosol, and another is membrane MR (mMRs). Classically, corticosterone acts through gMRs, which as transcription factors and mediate various gene expression pathways. In contrast, corticosterone acts on mMRs through signal transduction that enables rapid modulation of synaptic transmission and facilitates the regulation of membrane ionic currents.^[^ ^48^, ^51^, ^52^, ^53^ ^]^ Interestingly, our results also suggest that MR likely exerts its effects via mMRs, rather than gMR‐mediated transcriptional pathways. This is based on our observation that the inhibition of the synthesis of new proteins had no significant influence on MR‐induced enhancements in the presynaptic glutamate release and intrinsic excitability of BLA^→NAcC^ neurons (Figure S4, Supporting information). This supports the idea that corticosterone rapidly modulates BLA^→NAcC^ neurons function through mMR‐mediated signaling, consistent with prior reports implicating mMRs in fast, non‐genomic responses, including the modulation of presynaptic mGlu2 receptors.^[^ ^49^, ^54^, ^55^, ^56^ ^]^

Previous studies showed that corticosterone could impair retrieval of memory,^[^ ^57^, ^58^, ^59^, ^60^ ^]^ whereas the current research indicates that the context‐induced elevation in serum corticosterone promoted the retrieval of morphine withdrawal memories. Reasons for this difference may be related to the serum corticosterone levels achieved under these conditions and the receptors they act on. The concentration of serum corticosterone during the retrieval of morphine memory in our study was ≈194 ng ml^−1^, which is an intermediate level under low stress conditions and was close to that of 0.5 mg kg^−1^ corticosterone injection systemically to ADX mice.^[^ ^61^ ^]^ While in other studies, the concentration of corticosterone that impaired the retrieval of memory was higher (more than 300 ng ml^−1^), resembling to several stress conditions, and the dosage of corticosterone injected systemically was 1, 3, or 10 mg kg^−1^.^[^ ^62^, ^63^, ^64^ ^]^ Lower serum corticosterone may act mainly on the high‐affinity MR to induce a promotion of memory. The evidence supporting this statement was 1) Rimmele et al. reported that blocking MR impaired memory retrieval;^[^ ^46^ ^]^ 2) Our present result showed that MR in the BLA is involved in CTR‐MWM. Higher serum corticosterone may act mainly on the low‐affinity GR to inhibit memory retrieval. The evidence supporting this statement was 1) the activation of GR impaired memory retrieval;^[^ ^65^, ^66^ ^]^ 2) blockade of GR enhanced memory retrieval.^[^ ^46^ ^]^ This is consistent with the classic theory that corticosterone exhibits an inverted U‐shaped correlation with memory retrieval.^[^ ^67^ ^]^

In conclusion, the current research indicates that corticosterone contributes to CTR‐MWM by acting on BLA^→NAcC^ neurons, and the modulation of corticosterone pathways is important for the development of therapies for drug relapse.

## 4 Experimental Section

### Animals

Male C57BL/6J mice (6‐12 weeks, Lingchang Biotechnology, Shanghai) were kept in a 12‐h light cycle with regulated humidity and temperature. They have unlimited access to food and drink. Every experimental methodology complied with worldwide animal welfare standards and Fudan University's ethical guidelines (20220303‐006). The study used a minimal number of animals with an attempt to reduce their suffering.

### Stereotaxic Surgery and Microinjection

Mice were anesthetized through intraperitoneal administration of 1.2% Avertin (200 mg kg^−1^). For retrograde labeling experiments, CTB‐555 (#C34776, Invitrogen, USA) or FluoSpheres‐505/515 (#F8859, Invitrogen, USA) was microinjected into the NAcC (0.3 µL, 1.58 mm posterior to bregma, 1.22 mm lateral, 4.57 mm ventral). AAV‐hSyn‐mCherry‐WGA‐Cre (4.28E + 12 vg ml^−1^; Neuron Biotech Company, China) was microinjected into the NAcC, and AAV‐DIO‐hM4D(i)‐EGFP (3.44E + 12 vg ml^−1^; Neuron Biotech Company, China) was microinjected into the BLA, to investigate in vivo chemogenetic regulation in our behavioral paradigm. For RNA interference silencing experiments, retro‐AAV‐Cre‐mCherry (5.80E + 12 vg ml^−1^ ; BrainVTA, China) was microinjected into the NAcC and AAV‐hSyn‐DIO‐shRNA (Nr3c2/scramble; 5.13E + 12 vg ml^−1^; BrainVTA, China) was microinjected into the BLA. A 10‐min post‐infusion retention period was implemented for all stereotaxic procedures to ensure proper drug delivery. For cannula placement, guide cannulas (RWD Life Science, China) were aimed 1 mm above the BLA. Corticosterone (30 nm; MCE, USA) was administrated into BLA (0.3 µL) 15 min before the post‐test. Mifepristone (30 ng; Sigma, USA) was administrated into BLA (0.3 µL) 20 min before the post‐test. Spironolactone (50 ng; MCE, USA) was administrated into BLA (0.3 µL) 20 min before the post‐test. SKF38393 (1 µg; MCE, USA) was administrated into BLA (0.3 µL) 10 min before the post‐test. To prevent corneal drying, aureomycin eye ointment was applied, and a heat pad maintained ≈37 °C was used during surgeries. Mice for CTB‐555 or FluoSpheres‐505/515 injection and cannula placement were allowed 1 week for recuperation before the behavioral assay, and mice injected with virus were allowed 3–4 weeks for recuperation and virus expression. Animals with missed‐site injection or placement were included in this study via preparing coronal sections.

### Conditioned Place Aversion (CPA)

The CPA paradigm was performed as previously described, utilizing a Med Associates apparatus featuring different tactile and visual contexts.^[^ ^27^, ^40^, ^68^, ^69^ ^]^ The CPA protocol includes four distinct phases: on Day 1 of *Pre‐test*: mice were placed in the central neutral chamber for 15 min freely exploration in the apparatus. Mice exhibiting a strong unconditioned preference (> 80%) for any chamber were excluded. Eligible mice were randomized to four groups: SS, MS, SN, and MN. Day 2–6 of *Drug Treatment*: morphine dependency was established in the MS and MN groups through a systematic intraperitoneal injection regimen of morphine (Northeast Pharmaceutical Group, China), administered twice daily at 8:30 and 18:30 h. The dosage (mg kg^−1^) was increased over a 5‐day period: 10 on Day 1, 20 on Day 2, 30 on Day 3, and 40 on Day 4 and 5. Mice in the SS and SN groups received equivalent volumes of saline. All administrations were performed in the animals’ home cages. Day 7–10 of *Conditioning*: on Day 7 and 9, mice in the MN group were intraperitoneally injected with naloxone (0.3 mg kg^−1^; #S3066, Selleckchem, USA) 2 h after morphine administration (40 mg kg^−1^) to precipitate withdrawal. They were then restricted to their withdrawal‐associated chamber (the slightly preferred chamber during the pre‐test) for 20 min; on Day 8 and 10, mice in the MN group were intraperitoneally injected with saline 2 h after morphine administration (40 mg kg^−1^) and restricted to their saline‐associated chamber (the opposite chamber) for 20 min. Day 11 of *Post‐test*: mice were placed in the central neutral chamber for 15 min freely exploration in the apparatus 24 h after the final conditioning session on Day 10. In the pre‐test, the CPA score was calculated as the duration in the slight preferred chamber minus that in the opposite chamber. Given that the conditioning of withdrawal occurred in the slightly preferred chamber, this place preference would shift to the opposite after two withdrawal training sessions. Mice in the control groups (SS, MS, and SN) underwent the same CPA procedures except for the administration regimen; mice in the NR group underwent the same CPA procedures as the MN group, except for post‐test, during which mice remained in their home cage with no stress (Figure 1A). For DREADD experiments, vehicle or CNO (1 mg kg^−1^, #S6887, Selleckchem) was administered 45 min prior to the post‐test.

### Corticosterone Assay

Trunk blood, from mice deeply anesthetized by inhaled isoflurane (> 5%) and decapitated rapidly after the post‐test, was collected into tubes and left to clot (20 min, room temperature). Then the samples were centrifuged to isolate the serum (10 min, 4 °C, 1700 × g) and kept at −80 °C until assay. Blood collection was performed between 14:00‐16:00 PM to exclude the effect of circadian rhythm on corticosterone concentration. The concentration of corticosterone was determined using an ELISA kit (#K014 – H1; Arbor Assays, USA) following the manufacturer's instructions precisely.

### Adrenalectomy

Bilateral ADX was conducted following an established surgical protocol with minor modification.^[^ ^70^, ^71^, ^72^ ^]^ In short, mice were intraperitoneally anesthetized by 1.2% Avertin (200 mg kg^−1^) and fixed on the surgical bed in a lateral position. Bilateral adrenal glands were bluntly isolated and then removed using a small curved forceps and a micro scissor. After surgery, mice were removed to a heat pad at 37 °C until waking up from anesthesia and were given 0.9% saline solution through the experiments. Mice in the sham group were subjected to an identical procedure but with the adrenal gland intact.

### Immunofluorescence and Imaging

Following a 90‐min post‐test interval, mice underwent transcardial perfusion with saline and were fixed in 4% paraformaldehyde for 16 h. Coronal slices were made for analysis using a vibratome (40 µm; Leica, USA) and then blocked at 37 °C for 2 h in phosphate‐buffered saline (PBS) containing 0.3% Triton X‐100 and 10% goat serum. Sections were incubated with primary antibody of c‐Fos (1:1000 dilution; #226005, Synaptic Systems, Germany) or MR (1:250 dilution; #ab64457, Abcam, UK) at 4 °C for 16 h, followed by incubation with biotinylated secondary antibody (1:500 dilution, #BA‐7000 or #BA‐1000, Vector, USA;) and Streptavidin conjugation (1:1000 dilution; #405235 or #405237, Biolegend, USA) at 37 °C for 1 h separately, and DAPI (1:10000 dilution; #C1002, Beyotime, China) at 37 °C for 8 min in the end. Following final PBS washes, sections were mounted and imaged using confocal microscopy. Quantitative analysis was performed using ImageJ software with consistent threshold parameters, where positive cells were identified as those exhibiting immunoreactivity exceeding basal background levels. Cell counts from at least four representative sections per animal were averaged to generate an individual value.

### Western Blot

Proteins of BLA were extracted using RIPA Lysis Buffer (P0013B, Beyotime Biotechnology, China) with 1% Protease Inhibitor Cocktail (20124ES03, Yeasen, China) and 1% Phosphate Inhibitor Cocktail (20109ES05, Yeasen, China), homogenized 45 s in 70 Hz twice. Proteins were separated with SDS–PAGE and then transferred to nitrocellulose membranes. After blocking with Western Blocking Buffer (P0023B, Beyotime Biotechnology, China), membranes were incubated with primary antibodies (MR: 1:500, DF13302, Affinity Biosciences, AUS; β‐Actin: 1:1000, sc‐47778, Santa Cruz Biotechnology, USA) at 4 °C for 16 h and secondary antibodies (1:10000, 926–68021 and 926–32210, LI‐COR Biosciences, USA) at room temperature for 1 h. Membranes were visualized using an Odyssey infrared imaging system (LI‐COR Biosciences, USA). Quantitative analysis was performed using Image J software.

### Electrophysiological Recording

250 µm brain slices containing BLA were made in ice‐cold and oxygenated artificial cerebrospinal fluid (ACSF) with a vibratome (Leica, GER). The ACSF had the following composition (in mm): 92 NaCl, 2.5 KCl, 4 MgSO_4_, 1.5 NaH_2_PO_4_, 30 NaHCO_3_, 25 glucose, 10 HEPES, 2 thiourea, 3 sodium pyruvate, and 2 CaCl_2_ (pH = 7.35). Following preparation, brain slices were maintained in oxygenated ACSF 1 h prior to electrophysiological recordings at 32 °C. Whole‐cell patch‐clamp recordings were performed using an EPC10 amplifier controlled by PatchMaster 2.54 software (HEKA, Lambrecht, GER). Recording pipettes (3–5 MΩ resistance) were filled with intracellular solution containing (in mm): 140 K‐Gluconate, 0.1 CaCl_2_, 2 MgCl_2_, 1 EGTA, 2 K_2_‐ATP, 0.1 Na_3_‐ATP, 10 HEPES (pH = 7.35, adjusted with KOH). BLA^→NAcC^ neurons were identified with differential interference contrast and fluorescent microscopy. mEPSCs were recorded in the voltage‐clamp mode (holding at −70 mV) with 1 µm TTX, 50 µm APV, and 10 µm SR95531. Increasing current steps‐induced AP firing was assessed in the current‐clamp mode (holding at 0 pA; current steps: 0–350 pA in 50 pA increments) with 50 µm APV, 10 µm SR95531, and 10 µm CNQX. AHP measurements were conducted as follows: thefAHP was measured between the second and third evokes spikes as the difference between the peak of the spike and the threshold (cells with less than three spikes were excluded); the mAHP was measured as the difference between the peak of AHP following the current pulse and the baseline membrane potential; the sAHP was measured as the difference between the average membrane potential from 280 to 320 ms after the current pulse and the baseline membrane potential.^[^ ^73^, ^74^, ^75^ ^]^

### Statistical Analysis

All data analyses and graphical representations were conducted with GraphPad Prism (V8.0). For comparisons involving two groups, Student's *t*‐test was employed if the data were normally distributed, and the Wilcoxon test was employed if not. When comparing three or more groups, one‐way or two‐way analysis of variance (ANOVA) followed by Bonferroni's multiple comparisons test was utilized if the data were normally distributed, and the Friedman test was employed if not. The sample size (n) represents either the number of experimental animals or recorded cells. The data of electrophysiological experiments were analyzed using Mini‐Analysis (Synaptosoft) and Clampfit (Axon Instruments) software. Data were presented as mean ± standard error of the mean (SEM) throughout the study.

## Conflict of Interest

The authors declare no conflict of interest.

## Supporting information



Supporting Information

## Data Availability

The data that support the findings of this study are available from the corresponding author upon reasonable request.
