# Supplementary material for: Corticosterone Contributes to Context‐Triggered Retrieval of Morphine Withdrawal Memories by Acting on Basolateral Amygdala Neurons Projecting to Nucleus Accumbens Core
Source: Adv Sci (Weinh). 2025 Aug 23;12(42):e03409. doi: 10.1002/advs.202503409 (PMC12622562; doi:10.1002/advs.202503409)
Supplement: Supplementary file 1 — Supporting Information [file ADVS-12-e03409-s001.docx]

Supporting Information

Corticosterone Contributes to Context-triggered Retrieval of Morphine Withdrawal Memories by Acting on Basolateral Amygdala Neurons Projecting to Nucleus Accumbens Core

Zixuan Cao, Yaxian Wen, Yuanqi Chen, Yali Fu, Hao Yang, Chenshan Chu, Xinli Guo, Yu Yuan, Chao Lei, Huan Sheng, Da Shao, Li Yang, Dongyang Cui, Ming Chen^*^, Bin Lai^*^, Ping Zheng^*^

**F****igure S1**

**
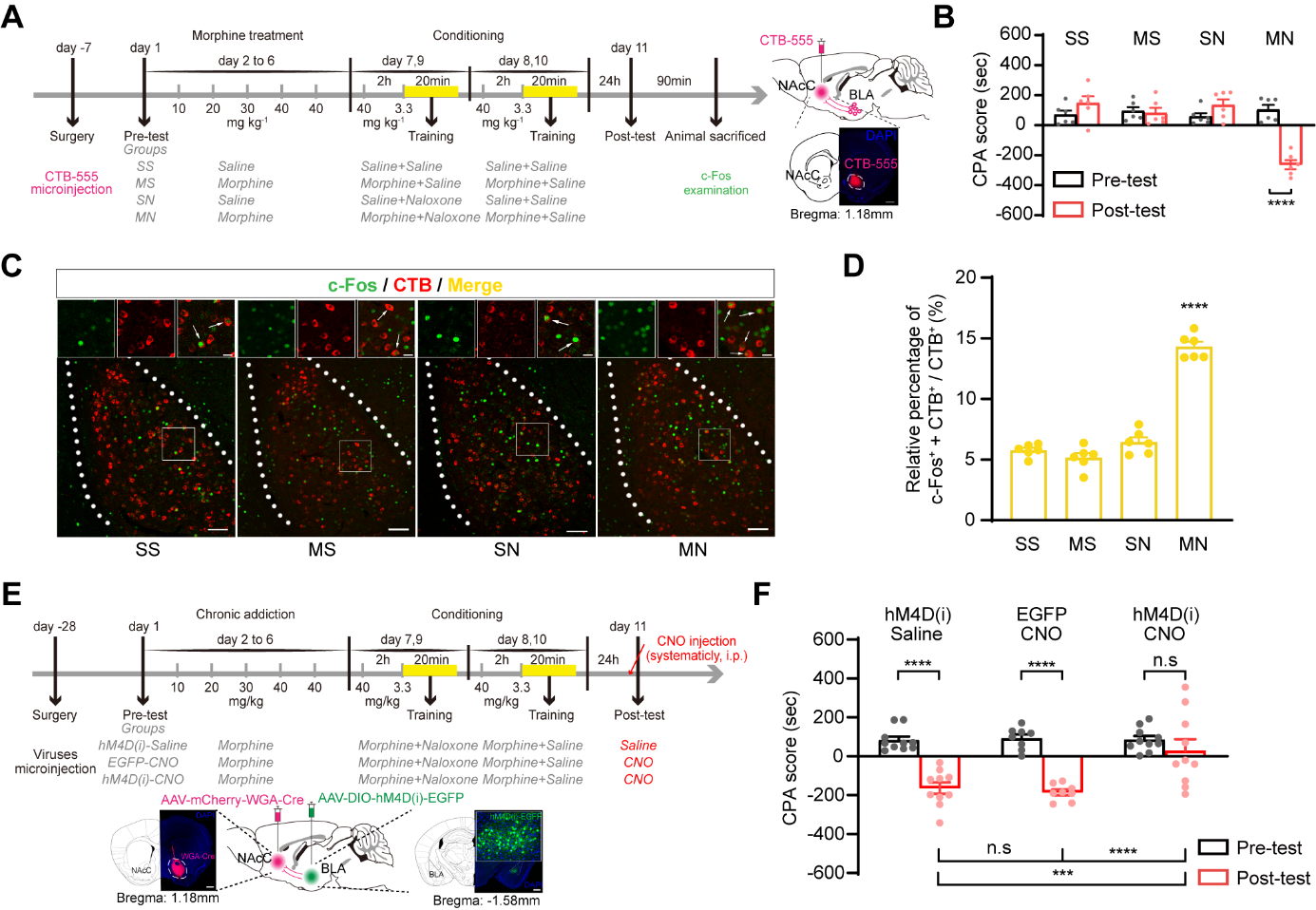
**

**Figure S1. The role of** **BLA^→NAcC^ neurons in CTR-MWM.** **(A).** Experimental scheme, diagram and anatomical location of the microinjection site of CTB-555 in NAcC. Scale bars, 500 μm. **(B).** The average CPA scores in SS, MS, SN and MN groups (n = 6 mice per group). **(C).** Immunofluorescence analysis of c-Fos^+^ (green) + CTB-555^+^ (red) co-labeled neurons in BLA. Magnified images show the boxed area. Scale bars, 100 and 20 μm. **(D).** The average proportion of c-Fos^+^ + CTB-555^+^ in CTB-555^+^ in BLA^→NAcC^ neurons (n = 6 mice per group). **(E)**. Experimental scheme, diagram of microinjection and the expression of virus. Scale bar, 500 μm. **(F).** The average CPA scores in the hM4Di + Sal (n = 10 mice) group, EGFP + CNO (n = 8 mice) group and hM4Di + CNO (n = 10 mice) group. ^***^P < 0.001, ^****^P < 0.0001. Means ± SEMs.

**Figure S2
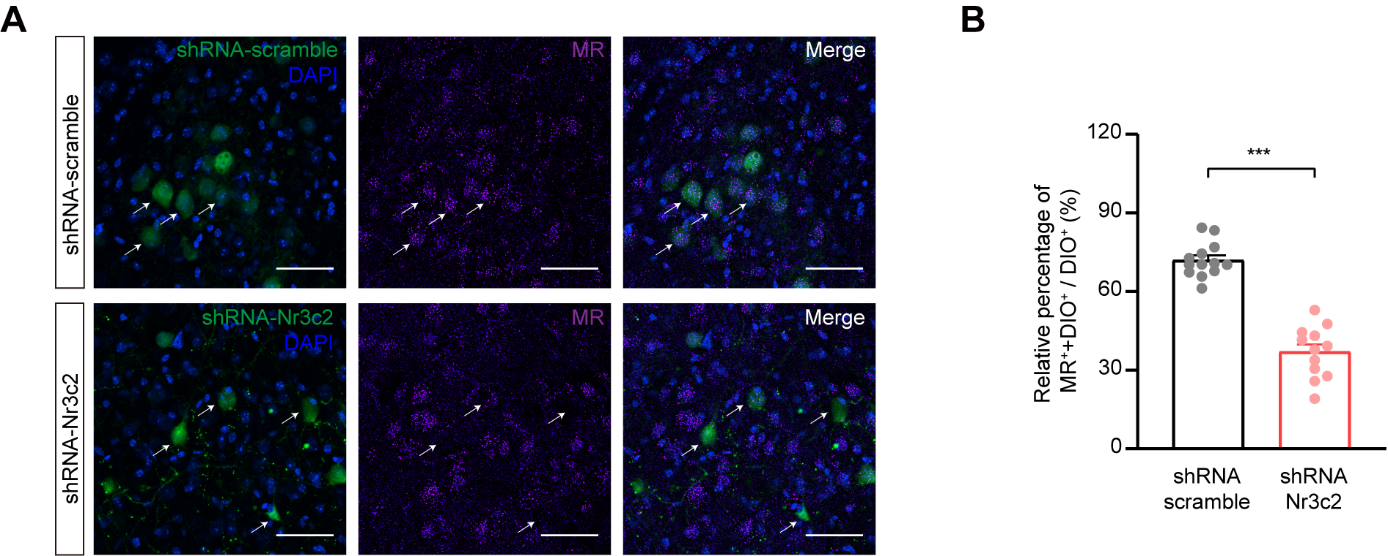
**

**Figure S2. The relative expression levels of MR in** **BLA^→NAcC^ neurons from shRNA-scramble and shRNA-Nr3c2 mice.** **(A).** Immunofluorescence of MR^+^ (purple) + DIO^+^ (green) co-labeled neurons in BLA^→NAcC^ neurons. Scale bars, 50 μm. **(B).** The average relative percentage of MR^+^ (purple) + DIO^+^ (green) co-labeled neurons in BLA^→NAcC^ neurons in the shRNA-scramble (n = 13 samples from 3 mice) and shRNA-Nr3c2 groups (n = 12 samples from 5 mice). ^****^P < 0.0001. Means ± SEMs.

**Figure S3
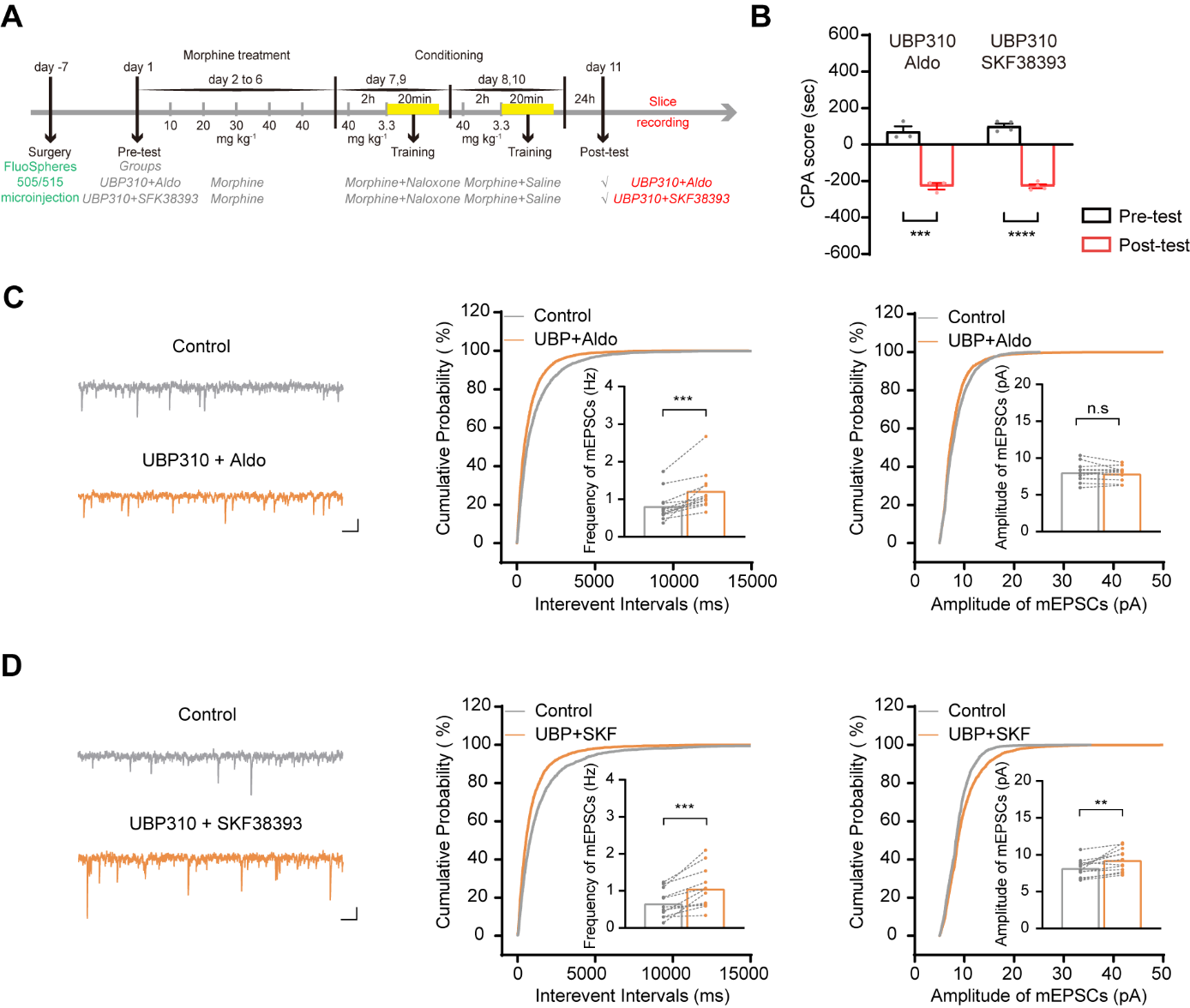
**

**Figure S3. The KA-independent effect of aldosterone and SKF38393 on the mEPSCs of BLA^→NAcC^ neurons during CTR-MWM. (A).** Experimental scheme. **(B).** The average CPA scores in the UBP310 + Aldo (n = 3 mice) and UBP310 + SKF38393 groups (n = 4 mice). **(C).** Representative traces, graph and probability cumulative curves of mEPSCs frequency and amplitude before (Control) and after UBP310 + Aldo treatment (n = 12 cells). Scale bars, 5 pA, 200 ms. **(D).** Representative traces, graph and probability cumulative curves of mEPSCs frequency and amplitude before (Control) and after UBP310 + SKF38393 treatment (n = 12 cells). Scale bars, 5 pA, 200 ms. ^**^P < 0.01, ^***^P < 0.001. Means ± SEMs.

**
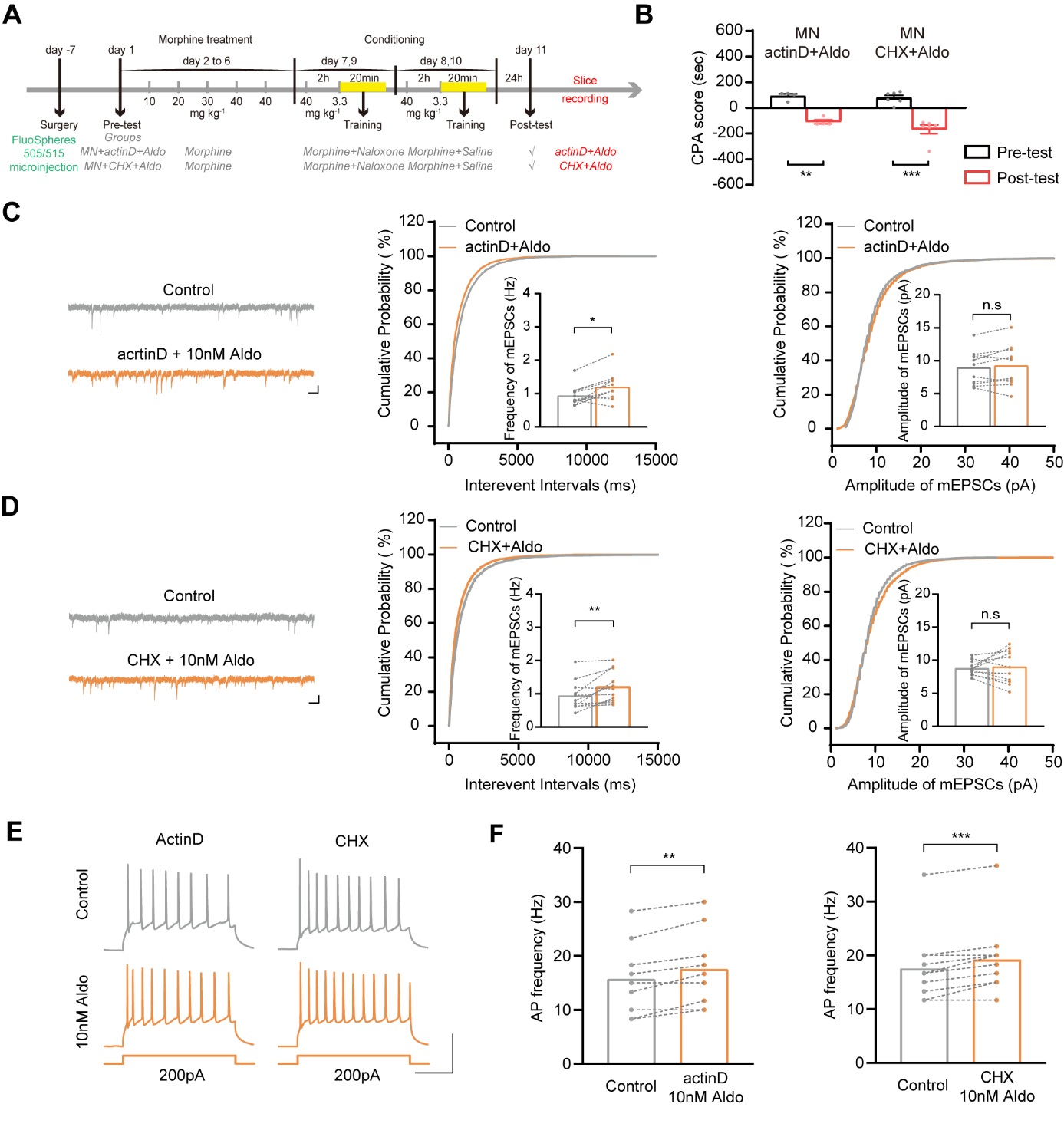
Figure S4**

**Figure S4. The nongenomic effect of aldosterone on the mEPSCs and AP frequency of BLA^→NAcC^ neurons during** **CTR-MWM. (A).** Experimental scheme. **(B).** The average CPA scores in the MN + actinD + Aldo (n = 4) and MN + CHX + Aldo groups (n = 6 mice). **(C).** Representative traces, graph and probability cumulative curves of mEPSCs frequency and amplitude before (Control) and after actinD + Aldo treatment (n = 11 cells from 4 mice). Scale bars, 5 pA, 200 ms. **(D).** Representative traces, graph and probability cumulative curves of mEPSCs frequency and amplitude before (Control) and after CHX + Aldo treatment (n = 11 cells from 6 mice). Scale bars, 5 pA, 200 ms. **(E).** Typical AP traces in response to 200 pA depolarizing currents before (Control) and after actinD + Aldo (*left*) or CHX + Aldo (*right*) treatment. Scale bars, 50 mV, 200 ms. **(F).** *Left*: the graph of AP frequency before (Control) and after actinD + Aldo treatment (n = 9 cells from 4 mice). *Right*: the graph of AP frequency before (Control) and after CHX + Aldo treatment (n = 11 cells from 6 mice). ^*^P < 0.05, ^**^P < 0.01, ^***^P < 0.001. Means ± SEMs.
